# The Relationship between the IFNG (rs2430561) Polymorphism and Metabolic Syndrome in Perimenopausal Women

**DOI:** 10.3390/medicina56080384

**Published:** 2020-07-31

**Authors:** Daria Schneider-Matyka, Małgorzata Szkup, Aleksander Jerzy Owczarek, Marzanna Stanisławska, Anna Knyszyńska, Anna Lubkowska, Elżbieta Grochans, Anna Jurczak

**Affiliations:** 1 Department of Nursing, Pomeranian Medical University in Szczecin, Żołnierska Str. 48, 71-210 Szczecin, Poland; daria.schneidermatyka@gmail.com (D.S.-M.); stamarz@pum.edu.pl (M.S.); grochans@pum.edu.pl (E.G.); 2 Health Promotion and Obesity Management Unit, Department of Pathophysiology, Medical University of Silesia in Katowice, Medyków Str. 18, 40-752 Katowice, Poland; aowczarek@sum.edu.pl; 3 Department of Functional Diagnostics and Physical Medicine, Pomeranian Medical University in Szczecin, Żołnierska Str. 54, 71-210 Szczecin, Poland; anna.knyszynska@pum.edu.pl (A.K.); anna.lubkowska@pum.edu.pl (A.L.); 4 Department of Specialized Nursing, Pomeranian Medical University in Szczecin, Żołnierska Str. 48, 71-210 Szczecin, Poland; jurczaka@op.pl

**Keywords:** metabolic syndrome, *IFNG* polymorphism, women’s health, chronic inflammation

## Abstract

*Background and Objectives:* Metabolic syndrome (MetS) is a multiple risk factor for atherosclerosis, cardiovascular disease, type 2 diabetes and strokes. One-third of middle-age women are at risk of MetS, which predisposes them to type 2 diabetes and cardiovascular disease. Changes in the regulation of anti-inflammatory cytokines―which play an important role in pathologic processes―may contribute to inflammatory disorders. Cytokine polymorphisms are known to have an impact on gene expression. The purpose of this study was to search for the relationship between the *IFNG* polymorphisms and the levels of proinflammatory cytokines. *Materials and Methods:* This study, conducted in West Pomeranian Voivodeship, Poland, involved 416 women. Of these women, 33.6% of them had primary education, 44.8% lived in cities with a population of over 100,000, and 82.7% were married. Of the participants, 28.4% met the criteria for MetS. The study involved: interview performed to collect sociodemographic and medical data, anthropometric measurements, as well as venous blood collection for biochemical analysis, genetic testing and the measurement of inflammatory markers. *Results*: The link between the *IFNG* (rs2430561) polymorphism and serum PIC (proinflammatory cytokines) levels was tested with regard to MetS. In the MetS+ subgroup, the T/T and A/T genotypes of the *IFNG* gene were accompanied by higher IL-6 levels than in the MetS− subgroup. *Conclusion:* Our study has not confirmed a direct link between the *IFNG* polymorphisms and the levels of inflammatory biomarkers. Nevertheless, the T/T and A/T genotypes of the IFNG gene may predispose to elevated IL-6 levels.

## 1. Introduction

Metabolic syndrome (MetS) is a multiple risk factor for atherosclerosis, cardiovascular disease, type 2 diabetes and strokes. It is a worldwide health problem; whose incidence rate ranges from 13.8% to over 60% in different populations [1,2,3,4]. MetS components are abdominal obesity, elevated serum triglyceride (TG) and glucose levels, raised blood pressure and decreased levels of high-density lipoprotein cholesterol (HDL-C). A clinical diagnosis of MetS is made if at least three of these components are present [5,6].

As a result of reduced sex hormone secretion in the perimenopausal period―a natural consequence of gonadal dysfunction and aging― many women develop chronic diseases from the sixth decade onwards, which affects their quality of life. As defined by the stages of reproductive aging workshop (STRAW) criteria, perimenopause is the time between the first major changes in the length of the menstrual cycle (variations of the individual’s normal cycle length greater than seven days) and the period of 12 consecutive months without menstruation [7]. Potentially, hormone-reactive tissues, such as those making up the brain, bones, and the cardiovascular system become more susceptible to diseases [8]. The most common metabolic disorders in menopause are dyslipidemia, impaired glucose tolerance, insulin resistance, hyperinsulinemia and type 2 diabetes. Menopause is regarded as a risk factor for MetS, probably due to lower estrogen levels and higher insulin resistance [3,9,10]. Additionally, increased LDL (Low-Density Lipoprotein) and decreased HDL (High-Density Lipoprotein) levels are observed after menopause, owing to hyperandrogenism caused by ovarian insufficiency [11,12]. Irrespective of age, pre- and postmenopausal periods involve an increase in the levels of triglycerides and a decrease in the levels of HDL-C [13].

One-third of middle-age women are at risk of developing MetS, which predisposes them to type 2 diabetes and cardiovascular disease (CVD) [14,15,16].

Obesity, insulin resistance and type 2 diabetes entail chronic inflammation, resulting from abnormal levels of cytokines, acute phase reactants and other inflammatory signaling markers [17]. MetS is also believed to be associated with chronic inflammation characterized by greater cytokine production and activation of inflammatory signaling pathways. This has been confirmed by the analysis of the relationship between high levels of C-reactive protein (CRP) (a sensitive marker of subclinical inflammation) and insulin resistance, as well as the components of Mets [18]. Biologic dysfunction in older postmenopausal women is related to ‘senile inflammation’, which shows a strong relationship between aging, inflammation and menopause [19]. The measurement of CRP, interleukin-6 (IL-6), IL-1 receptor antagonist (IL-1ra), IL-18, tumor necrosis factor alpha (TNFα)-R1, adiponectin, resistin, and leptin revealed that high levels of cytokines increase the likelihood of MetS in obese and non-obese individuals, irrespective of insulin resistance. Both non-obese and obese people with severe inflammation seem to be more likely to develop MetS than those with lower levels of inflammatory markers [20].

Changes in the regulation of anti-inflammatory cytokines―which play an important role in pathologic processes―may contribute to inflammatory disorders. Cytokine polymorphisms are known to have an impact on gene expression. Interferon gamma (IFNγ) is the main proinflammatory cytokine. Studies show that the *IFNG* +874 A/T single nucleotide polymorphism (SNP) located at the position +874 of intron 1 can affect the secretion of IFNγ. The IFNγ production is determined by the polymorphic *IFNG* (rs2430561) gene, which includes the T allele, contributing to high IFNγ production and the A allele responsible for low IFNγ production. Average levels of IFNγ cytokines are higher in healthy carriers of the T allele than in those with the A allele. It has been clearly demonstrated that the T-to-A polymorphism has a direct effect on the level of IFNγ production [21].

The purpose of this study was to analyze the relationship between the *IFNG* polymorphisms and the levels of proinflammatory cytokines (IL-1α, IL-1β, IL-6, TNFα, IFNγ) in women aged 45–60 years.

## 2. Material and Methods

This study, conducted in West Pomeranian Voivodeship (Poland), involved 416 women, whose mean age was 53.5 years. The inclusion criteria were female sex, the age of 45–60 years, using MHT and no history of inflammatory, psychiatric or neoplastic diseases. All participants were non-smokers, drank less than 20 g of pure alcohol per day or drank occasionally no more than 40 g of pure alcohol and abstained from alcohol at least two days per week. Of the participants, 33.6% had primary education, 27.7% had tertiary education, 44.8% lived in cities with a population of over 100,000, almost 40% lived in rural areas, 82.7% of the women were married, 28.4% met the criteria for MetS. Each participant gave informed written consent to take part in this study.

The study was carried out in accordance with ethical standards and the Declaration of Helsinki. The protocol was approved by the bioethical committee of the Pomeranian Medical University, Szczecin, Poland on 19 June 2017 (approval number KB-0012/181/13).

The study involved: interview performed to collect sociodemographic and medical data (hypertension, hypertriglyceridemia, hyperglycemia, elevated HDL levels); anthropometric measurements (blood pressure was determined by nurses following the binding procedures; waist size was measured in a standing position between the lower margin of the ribs and the upper margin of the iliac crest at the end of a gentle exhalation); venous blood collection for biochemical analysis, genetic testing and the measurement of inflammatory markers in accordance with the procedures for sampling and transport of the biologic material. Blood was taken between 7.00 and 9.30 a.m. after overnight fasting and ten-minute rest in a sitting position. Blood was collected to two Vacutainer tubes (Sarstedt, Nümbrecht, Germany): a tube containing 1-g/L dipotassium (K2) ethylenediaminetetraacetic acid (EDTA) and a tube for biochemical serum analysis (7 mL). The levels of triglycerides, HDL and glycemia after overnight fasting were determined. Next, DNA was isolated for genetic analysis of the *IFNG* (rs2430561) polymorphism. The biologic material for DNA analysis was stored and transported in accordance with the quality management system of the Genetic Laboratory, Clinic and Department of Psychiatry (according to standard EN 15,189).

Additionally, the levels of inflammatory markers (IL-1α, IL-1β IL-6, TNFα, IFNγ) were determined.

Based on the results of laboratory and anthropometric measurements, the women were qualified to two subgroups: the MetS+ subgroup (*n* = 118) comprising respondents who met the criteria for MetS based on the International diabetes federation (IDF) classification [22] and the MetS-subgroup (*n* = 298)—including those who did not meet the above-mentioned criteria. The respondents were assigned to the MetS+ subgroup if they had at least three out of the five components of MetS: waist size ≥80 cm; glycemia on an empty overnight fasting ≥100 mg/dL (5.6 mmol/L) or related pharmacotherapy; the levels of triglycerides ≥150 mg/dL (1.7 mmol/L) or related pharmacotherapy; HDL cholesterol ≤50 mg/dL (1.3 mmol/L) or related pharmacotherapy; systolic blood pressure ≥130 and/or diastolic blood pressure ≥85 mmHg or related pharmacotherapy.

Statistical analysis was performed using Statistica 13.0 PL (TIBCO, Palo Alto, CA, USA) and R software (R Core Team (2013). R: A language and environment for statistical computing. R Foundation for Statistical Computing, Vienna, Austria). Statistical significance was set as *p* < 0.05. All tests were two-tailed. The two groups (MetS+ and MetS−) were compared using the Student’s *t-*test in the case of normal data distribution and the Mann–Whitney U test in the case of skewed data distribution. To assess the link between MetS and particular genotypes, four inheritance models were tested with the Bayesian information criterion (BIC) in order to select the best one. An effect of particular variables and alleles on the development of MetS was calculated using odds ratio (OR) with 95% confidence interval (CI).

The results presented in this manuscript are a part of the larger project performed by a team of researchers.

## 3. Results

In the study sample, we observed a statistically significant relationship (*p* < 0.001) between waist size, the levels of glycemia after overnight fasting, triglycerides and cholesterol, systolic and diastolic blood pressure and MetS. A statistically significant relationship (*p* < 0.05) was also noticed between Mets and the level of IL-6. MetS was not statistically significantly related to the levels of IL-1α, IL-1β, TNFα and IFNγ (Table 1).

We analyzed the distribution of the genotypes and alleles of the *IFNG* (rs2430561) polymorphism with regard to MetS. The T/T genotype was found in 45% of the participants, the A/T genotype in 39% and the A/A genotype in 16%. The T allele was more common (64%) than the A allele (36%). There were no significant differences in the distribution of the genotypes and alleles between the participants who met the criteria for MetS and those who did not. We tested the link between the *IFNG* (rs2430561) polymorphism and serum PIC levels with regard to MetS. In the MetS+ subgroup, the T/T and A/T genotypes of the *IFNG* gene were accompanied by higher IL-6 levels than in the MetS− subgroup.

The levels of IL-1α, IL-1β, TNFα and IFN-γ were not statistically significantly related to any of the tested genotypes of the *IFNG* gene in any of the MetS subgroups (Table 2).

## 4. Discussion

Our study demonstrated that in the MetS+ subgroup, the presence of the T/T and A/T genotypes of the *IFNG* gene was associated with higher IL-6 levels than in the MetS− subgroup. The levels of IL-1α, IL-1β, TNFα and IFN-γ were not related to any of the tested *IFNG* genotypes.

*De Oliveira Silva* et al. who analyzed inflammatory parameters (IL-6, CRP, TNF-α, IFN-γ) in older women with and without MetS, found that those with MetS had higher levels of cytokines and risk factors for cardiovascular disease (the correlations were weak, but significant) [13]. Other authors compared a group of 200 patients with type 2 diabetes and 100 healthy respondents from the control group. They determined anthropometric parameters, lipid parameters and the levels of CRP and cytokines (including IL-10, TNF-α and IFN-γ). The levels of cytokines were found to be higher in people with type 2 diabetes, however, it was not closely related to Mets. The authors observed limited correlations between each of the cytokines and Mets parameters [23]. In a study of 367 Mexican–Americans, type 2 diabetes was strongly related to elevated levels of IL-6, leptin, CRP and TNF-α, whereas higher glucose levels correlated positively and linearly with high levels of IL-6 and leptin [24].

In our study, MetS was significantly related to the levels of IL-6, but not to the levels of IL-1α, IL-1β, TNFα and IFNγ. The results show that the *IFNG* +874 T polymorphism has an effect on the expression of this gene. The A/A genotype was accompanied by lower levels of IFNγ than the T/T genotype and the heterozygous T/A genotype entailed average IFNγ levels [11]. IFNγ takes part in the pathogenesis of diabetes, and the frequency of the low IFNγ production allele (the A allele) was considerably higher in patients with type 2 diabetes compared with the control group [25]. Polymorphisms of genes encoding pro- and anti-inflammatory cytokines (TNFα-308 G/A, IFNγ +874 A/T, IL-10–1082G) may be responsible for nerve damage in neuropathy, which is a common complication of diabetes. The high IL-10 production -1082 G/G genotype and the low IFNγ production +874 A/A genotype may be liable for the weakening of the immune response, leading to inflammation [26]. It was also found that the T/A genotype of the *IFNG* +874 T/A polymorphism was linked to lower levels of total cholesterol and triglycerides [27].

We analyzed the influence of the *IFNG* +874 polymorphism on the levels of selected inflammatory biomarkers in patients with and without MetS. Respondents having no history of inflammatory, psychiatric or cancerous diseases and not using MHT were qualified to participate in the study. All participants were non-smokers and did not drink excess alcohol. The results did not reveal significant differences in the distribution of genotypes and alleles between the Mets+ and the Mets− subgroups. However, in the MetS+ subgroup, the T/T and A/T genotypes of the *IFNG* gene were accompanied by higher IL-6 levels than in the MetS− subgroup. There were no significant differences in the levels of other biomarkers (IL-1α, IL-1β, TNFα, IFNγ).

The study presented here has some limitations. Due to the substantial influence of sociodemographic factors and health behaviors on the respondents’ health status, we are not able to clearly determine the influence of the T/T and A/T genotypes of the *IFNG* gene on the level of IL-6. The study by Kim et al. demonstrated that the risk of cardiovascular disease, estimated using the Framingham risk score (FRS ≥ 10%), was associated with the socioeconomic status of the respondents more than with Mets [28]. Another limitation of our research is the lack of information on the respondents’ diet and the sources of nutritional products. The increased intake of carbohydrates with a high glycemic Index (GI) is known to directly induce insulin resistance and contribute to the development of type 2 diabetes in MetS patients. On the other hand, diets with a low GI, high in fiber, increase the feeling of satiety and reduce insulin resistance and the risk of type 2 diabetes [29]. It is also suggested that people’s health may be influenced by the local environment. Santulli et al. provided evidence that the use of the Mediterranean diet based on the short supply chain significantly reduces the incidence of MetS compared to the long supply chain. These authors indicate that the length of the food supply chain is a key determinant of the risk of MetS in the population living on a Mediterranean diet [30]. Furthermore, the study conducted by Assaf-Baut et al. among women with and without gestational diabetes mellitus (GDM) in the 12th–14th week after childbirth shows that the intervention on diet reduced the relative risk of MetS, but not the risk of insulin resistance [31].

## 5. Conclusions

Our study has not confirmed a direct link between the *IFNG* polymorphisms and the levels of the inflammatory biomarkers in perimenopausal women analyzed with regard to MetS. However, the presence of the T/T and A/T genotypes of the *IFNG* gene may predispose to elevated IL-6 levels.

## Figures and Tables

**Table 1:** Characteristics of the study sample with regard to a division into MetS+ and MetS− groups.

	MetS+ *N* = 118	MetS− *N* = 298	*p*
**MetS Components**			
waist size (cm)	93.3 ± 11.0	85.4 ± 11.2	**<0.001**
MetS symptom definition—waist size (cm); N (%)	97 (82.2)	62 (20.8)	**<0.001**
fasting glycemia (mg/dL)	100.8 (86.9–119.0)	83.2 (77.4–90.7)	**<0.001**
MetS symptom definition—hyperglycemia; N (%)	60 (50.8)	14 (4.7)	**<0.001**
TG (mg/dL)	137.6 (102.0–189.8)	84.8 (65.0–112.1)	**<0.001**
MetS symptom definition—TG; N (%)	75 (63.6)	32 (10.7)	**<0.001**
HDL (mg/dL)	56.5 ± 16.8	70.0 ± 16.0	**<0.001**
MetS symptom definition—HDL; N (%)	61 (51.7)	21 (7.1)	**<0.001**
systolic blood pressure (mmHg)	137.2 ± 15.3	119.1 ± 14.8	**<0.001**
diastolic blood pressure (mmHg)	83.9 ± 9.4	75.9 ± 9.7	**<0.001**
Mets symptom definition—hypertension; N (%)	97 (82.2)	62 (20.8)	**<0.001**
**PICs**			
IL-1α (pg/mL)	1.85 (1.46–2.42)	2.09 (1.50–2.59)	0.079
IL-1β (pg/mL)	13.64 (5.60–102.00)	12.39 (3.41–218.30)	0.801
IL-6 (pg/mL)	11.23 (5.51–34.47)	8.21 (3.65–21.46)	**<0.05**
TNFα (pg/mL)	4.06 (2.00–6.85)	0.05 (0.03–0.14)	0.495
IFNγ (IU/mL)	0.04 (0.03–0.21)	1.90 (1.30–3.20)	0.936

mean ± standard deviation; median (lower quartile–upper quartile); *p*—significance level. PICs—proinflammatory cytokines; CRP—C-reactive protein; IL—interleukin; TNFα—tumor necrosis factor α; IFNγ—interferon γ; MetS—Metabolic syndrome; TG—elevated serum triglyceride; HDL—high-density lipoprotein. Bold words shows that results are statistically significant.

**Table 2:** Analysis of the relationship between the *IFNG* (rs2430561) polymorphism and the levels of IL-1α, IL-1β, IL-6, TNFα and IFNγ with regard to MetS.

*IFNG* *Genotype*	MetS+	MetS−		
	Mean Value (SE); *N*	Mean Value (SE); *N*	Δ	±95% CI
log_10_ (IL-1α (pg/mL))				
T/T	0.311 (0.045); 56	0.397 (0.040); 132	−0.085	−0.210 ÷ 0.040
A/T	0.283 (0.027); 41	0.362 (0.033); 120	−0.079	−0.221 ÷ 0.063
A/A	0.385 (0.127); 21	0.369 (0.064); 46	0.016	−0.191 ÷ 0.222
log_10_ (IL-1β (pg/mL))				
T/T	1.209 (0.129); 47	1.242 (0.097); 107	−0.034	−0.387 ÷ 0.319
A/T	1.350 (0.155); 33	1.366 (0.110); 100	−0.016	−0.421 ÷ 0.389
A/A	1.593 (0.220); 20	1.408 (0.203); 20	0.185	−0.378 ÷ 0.748
log_10_ (IL-6 (pg/mL))				
T/T	**1.279 (0.103); 49**	**1.026 (0.059); 114**	**0.252**	**0.038 ÷ 0.467** *
A/T	**1.320 (0.119); 37**	**1.045 (0.061); 110**	**0.275**	**0.037 ÷ 0.514** *
A/A	0.915 (0.146); 2	0.893 (0.072); 42	0.021	−0.332 ÷ 0.375
log_10_ (TNFα (pg/mL))				
T/T	0.641 (0.079); 43	0.553 (0.047); 107	0.087	−0.078 ÷ 0.253
A/T	0.561 (0.072); 31	0.535(0.044); 105	0.026	−0.162 ÷ 0.213
A/A	0.599 (0.115); 17	0.518 (0.067); 41	0.081	−0.184 ÷ 0.346
log_10_ (INFγ (IU/mL))				
T/T	−1.048 (0.156); 23	−1.187 (0.054); 71	−0.138	−0.123 ÷ 0.400
A/T	−0.951 (0.169); 20	−1.077 (0.062); 67	0.126	−0.152 ÷ 0.404
A/A	−1.245 (0.126); 9	−1.040 (0.115); 27	−0.205	−0.625 ÷ 0.214

N—number of cases; SE—standard error of the mean; ∆—mean difference between groups; ±95% CI—95% confidence interval, IL—interleukin, TNFα—tumor necrosis factor α, IFNγ—interferon γ. *—statistically significant based on the ±95% CI. Bold words shows that results are statistically significant。
